# Albumin‐based hydrogels for regenerative engineering and cell transplantation

**DOI:** 10.1002/bit.27167

**Published:** 2019-10-06

**Authors:** John Ong, Junzhe Zhao, Alexander W. Justin, Athina E. Markaki

**Affiliations:** ^1^ Department of Engineering University of Cambridge Cambridge UK; ^2^ Gastroenterology Specialty Training Program East of England Deanery Cambridge UK

**Keywords:** crosslinking, hydrogel, regenerative medicine, serum albumin, stem cells, tissue engineering

## Abstract

Albumin, the most abundant plasma protein in mammals, is a versatile and easily obtainable biomaterial. It is pH and temperature responsive, dissolvable in high concentrations and gels readily in defined conditions. This versatility, together with its inexpensiveness and biocompatibility, makes albumin an attractive biomaterial for biomedical research and therapeutics. So far, clinical research in albumin has centered mainly on its use as a carrier molecule or nanoparticle to improve drug pharmacokinetics and delivery to target sites. In contrast, research in albumin‐based hydrogels is less established albeit growing in interest over recent years. In this minireview, we report current literature and critically discuss the synthesis, mechanical properties, biological effects and uses, biodegradability and cost of albumin hydrogels as a xeno‐free, customizable, and transplantable construct for tissue engineering and regenerative medicine.

## Graphical abstract

Albumin‐based hydrogels are under‐exploited biomaterials that are growing in interest. They hold great potential as xeno free, customisable and transplantable constructs for tissue engineering and regenerative medicine. This review discusses the synthesis, mechanical properties, biological effects and uses, biodegradability and cost of albumin hydrogels.

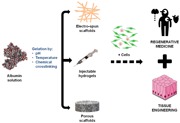

## 1 INTRODUCTION

Albumin, an endogenous, nonglycosylated protein, is produced predominantly in the liver by hepatocytes and secreted into the blood as a major constituent of plasma. It is comprised of 585 amino acids, has a molecular weight of 66.4 kDa, and an iso‐electric point of pH 4.7 (Vlasova & Saletsky, 2009). In vivo, albumin is a stable molecule because it is poorly metabolized, poorly immunogenic, and poorly filtered in the renal glomerulus (Lee & Youn, 2016). As a result, albumin has a physiological half‐life of approximately 19 days, during which it maintains oncotic pressure in the circulatory system, acts as a weak buffer, and stabilizes other important proteins, hormones, metal ions, nanoparticles, and drugs in vitro and in vivo. Albumin has two significant noncovalent binding sites that exogenous substances attach to, binding site 1 and 2. In so doing, the half‐life and treatment efficacy of drugs such as antibiotics, anti‐inflammatories, and synthetic insulin preparations are increased (Kratz, 2008; Lee & Youn, 2016). Other important biological characteristics of albumin include its accumulation at sites of inflammation from leaky capillaries and its active uptake by cancer cells, making it useful for targeting disease in molecular cancer therapeutics (Elsadek & Kratz, 2012; Lee & Youn, 2016).

Despite extensive research in albumin as a molecule for drug therapy, its use as a hydrogel in biomedical research is comparatively understudied. However, interest is steadily growing because albumin hydrogels offer a nonsynthetic, xeno‐free, and biocompatible biomaterial for the fields of tissue engineering and regenerative medicine which increasingly employ three‐dimensional (3D) cell cultures, tissue scaffolds, and constructs for disease modeling and transplantation. In addition, its inertness, stability, ability to gel at low concentrations, and the possibility of deriving patient‐specific albumin, make albumin hydrogels an attractive option. This review summarizes work on albumin hydrogels over the past decade and specifically discusses the (a) synthesis, (b) mechanical properties, (c) biological effects and uses, (d) biodegradability, and (e) cost.

For this review, the adopted definition of a hydrogel is a two‐ or multicomponent system, consisting of a 3D network of polymeric chains, where water occupies the spaces between those polymeric chains (Ahmed, 2015). Articles reporting hydrogels formed by other polymers but functionalized with albumin have been excluded in this review. A brief overview of the properties of albumin‐based hydrogels is provided in Table 1.

**Table 1:** Summary of albumin‐based hydrogel properties

Method of gelation vs. properties	pH‐induced albumin hydrogels	Thermally induced albumin hydrogels	Chemically crosslinked albumin hydrogels	References
Structure of albumin	pH < 2.3: E‐form (extended)	Variable: heat causes a range of changes from monomeric structural differences such as unfolding and disruption of secondary structure, dimerization, oligomerization, and polymerization	Dependent on crosslinking process and materials	Amiri, Jankeje, and Albani (2010); Barone et al. (1995); Chen et al. (2019); Leggio, Galantini, and Pavel (2008); Molodenskiy et al. (2017)
	pH 2.3–4.2: F‐form (fast migrating)			
	pH 4.3–8: N‐form (normal)			
	pH 8–10: B‐form (basic)			
	pH > 10: A‐form (aged)			
Mechanical properties				
Young's modulus	~46 kPa for 20 wt% BSA hydrogel, measured using indentation (Baler, Michael, Szleifer, and Ameer 2014)	~34 and ~67 kPa (pH 3.5), respectively, for 17 and 20 wt% BSA hydrogels, measured using indentation (Baler et al., 2014)	Not reported	Amdursky et al. (2018); Baler et al. (2014); Zhou et al. (2018)
		5–17 kPa for 3–9 wt% BSA hydrogels, measured under tension. Under confined compression, the values varied from ~0.2–4.4 kPa for 3–9 wt% BSA hydrogels (Amdursky et al., 2018)		
Storage G′ and loss modulus G″	G′ & G″: ~5–10 and ~60–80 kPa, respectively, for 16 and 20 wt% BSA hydrogels after 2,300 and 340 s (Baler et al., 2014)	G′ & G″: ~120 and ~60 kPa, respectively, for both 16 and 20 wt% BSA hydrogels (80°C, after 30–50 s; Baler et al., 2014)	Not reported	
		G′: 3–5 and 8–13 kPa, respectively, for 4.5 and 9 wt% BSA hydrogels. G″: 0.7–1.5 and 2–4 kPa, respectively, for 4.5 and 9 wt% BSA hydrogels (0.1–10 Hz; Amdursky et al., 2018)		
Tensile strength	Not reported	~2–5 MPa for 3–9 wt% BSA hydrogels (Amdursky et al., 2018)	~40 MPa for 10 wt% HSA hydrogel (Zhou et al., 2018)	
Hydrogel turbidity	Clear to translucent	Translucent to opaque (white). Highly dependent on ionic content, type of albumin, e.g., BSA vs. HSA, and albumin concentration	Clear to opaque; dependent on crosslinking process and materials	Amdursky et al. (2018); Arabi et al. (2018); Baler et al. (2014); Murata et al. (1993)
Biocompatibility	Cells cannot survive in the bulk of a strongly acidic or alkali hydrogel unless it is leached	Cells cannot survive the thermal gelation process	Almost all studies report good biocompatibility (cell survival and growth)	Baler et al. (2014); Hirose, Tachibana, and Tanabe (2010)
	Once leached, cells can be seeded on the surface or within pores of the hydrogel	Once gelled, hydrogels are biocompatible but cell attachment is often poor. Functionalisation of surfaces can be explored		
Biodegradability (duration)	Rapid: 1 day to 1 month	Long: >1 month	Variable: 2 weeks to >1 month	Baler et al. (2014); Feldman and McCauley (2018); Gallego, Junquera, Meana, García, and García (2010); Gallego, Junquera, Meana, Álvarez‐Viejo, and Fresno (2010); Kim et al. (2015); Raja, Thiruselvi, Mandal, and Gnanamani (2015)
Immunogenicity	Low	Low to moderate: fibrous capsule round transplanted scaffolds	Low to high: Dependent on crosslinking process and materials; for example, with glutaraldehyde, a fibrous capsule around the transplanted scaffold and evidence of local inflammation were noted. With PEG and species‐specific albumin, the above complications were absent	Amdursky et al. (2018); Baler et al. (2014); Feldman and McCauley (2018); Gallego, Junquera, Meana, García, and García (2010); Gallego, Junquera, Meana, Álvarez‐Viejo, and Fresno (2010); Kim et al. (2015); Ma et al. (2016); Raja et al. (2015)
Printability	Difficult: Low‐ and high‐pH albumin solutions are very viscous. Maybe problematic at high resolution and high pressures may be needed	Possible: High heat required to induce gelation of printed construct	Possible: Allows new gelation methods; for example, gelation by photo‐crosslinking of PEGDA‐albumin conjugates	No articles identified at the time of review
Current applications	Cardiac tissue engineering	Cardiac tissue engineering	Bone and cardiac tissue engineering, skin and wound healing, toxicology studies for liver disease models, stem cell‐derived nerve cells, drug delivery	See main text

Abbreviations: BSA, bovine serum albumin; HSA, human serum albumin; PEG, poly(ethylene glycol); PEGDA, poly(ethylene glycol) diacrylate.

## 2 SYNTHESIS OF ALBUMIN HYDROGELS

### 2.1 pH‐induced albumin hydrogels

**Figure 1:**
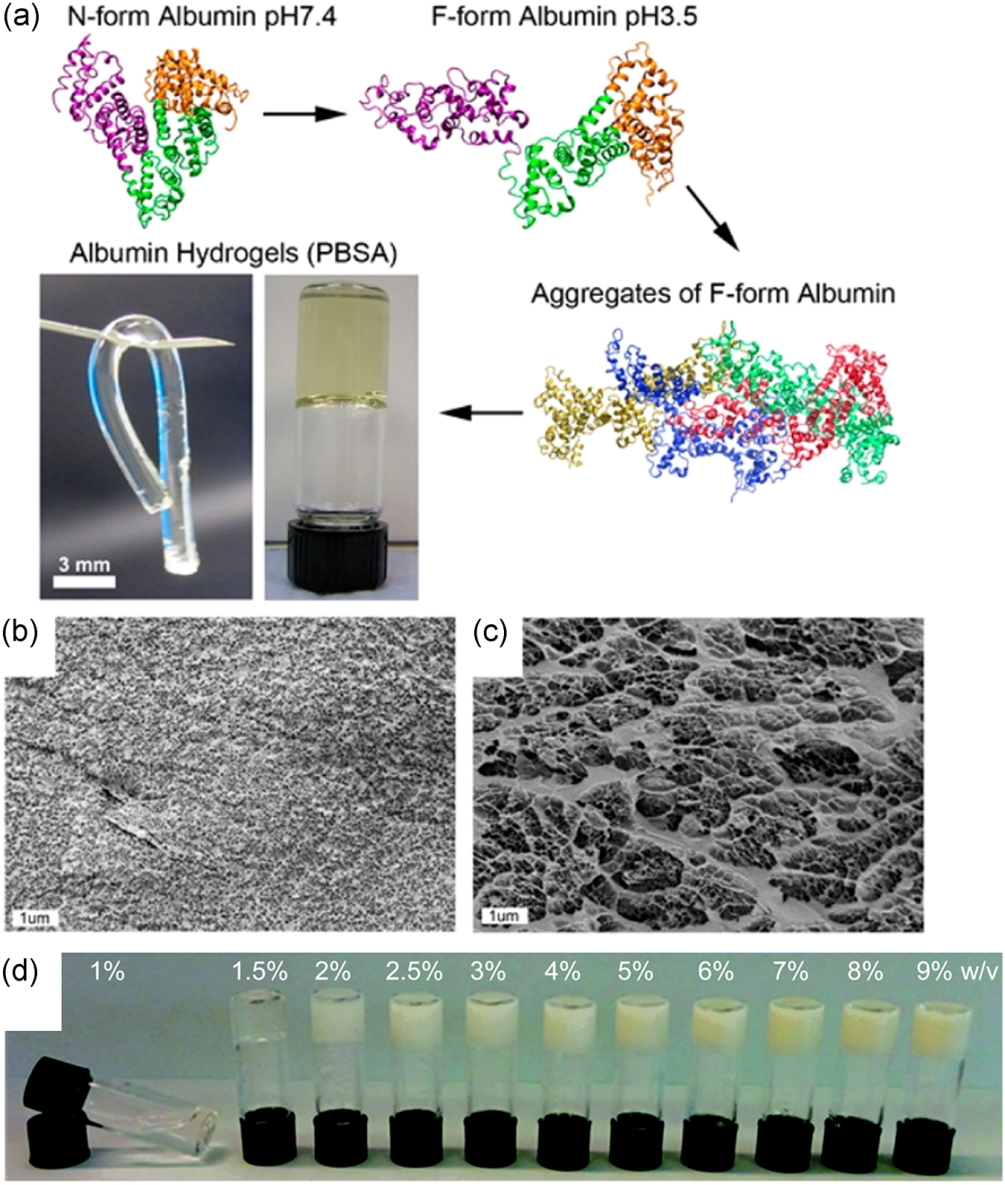
(a) Ribbon diagrams showing the partial denaturation of N‐form to F‐form albumin, protein aggregation, and hydrogel formation. Inverted vial shows a transparent pH‐induced BSA hydrogel (PBSA) next to a tubular PBSA cylinder made in mold at 37°C. Cryo‐SEM images of freeze‐fractured hydrogels formed at pH 3.5 at 37°C (b) and by thermally induced gelation at 80°C (c) illustrating differences in porosity. (d) Hydrogel turbidity of thermally induced BSA hydrogels increases with BSA concentration. BSA, bovine serum albumin; SEM, scanning electron microscope. Images (a–c) were reproduced with permission from Baler et al. (2014); https://pubs.acs.org/doi/abs/10.1021%2Facs.accounts.5b00438. Further permissions related to the material excerpted should be directed to the ACS. Image (d) was reproduced with permission from Amdursky et al. (2018)

Albumin exists either as monomers or oligomers depending on its environment (Barone et al., 1995; Molodenskiy et al., 2017). By manipulating pH, albumin in solution polymerizes and forms a clear hydrogel. Baler, Michael, Szleifer, and Ameer (2014) reported that by lowering the solution pH to 3.5 followed by 37°C incubation, bovine serum albumin (BSA) changes structure from the “N‐form” to the “F‐form” isomer, which then self‐assembles into a hydrogel network by hydrophobic interactions and counter ion binding (Figure 1). Crucially, neutralization of the acid‐induced hydrogels by leaching in Dulbecco's modified Eagle medium (DMEM) was required before acellular hydrogels could be transplanted into murine models. This implies it is not feasible to encapsulate pH‐sensitive cells in the bulk of the gel using this method of gelation. However, it does not preclude acid‐induced albumin hydrogels from being functionalized and used as a scaffold after pH neutralization is achieved. Also noteworthy is that BSA is only 76% similar in amino acid sequence compared to human serum albumin (HSA; Carter & Ho, 1994; X. M. He & Carter, 1992); therefore, gelation behavior and properties of HSA hydrogels may differ even if gelation methods and conditions are standardized.

**Figure 2:**
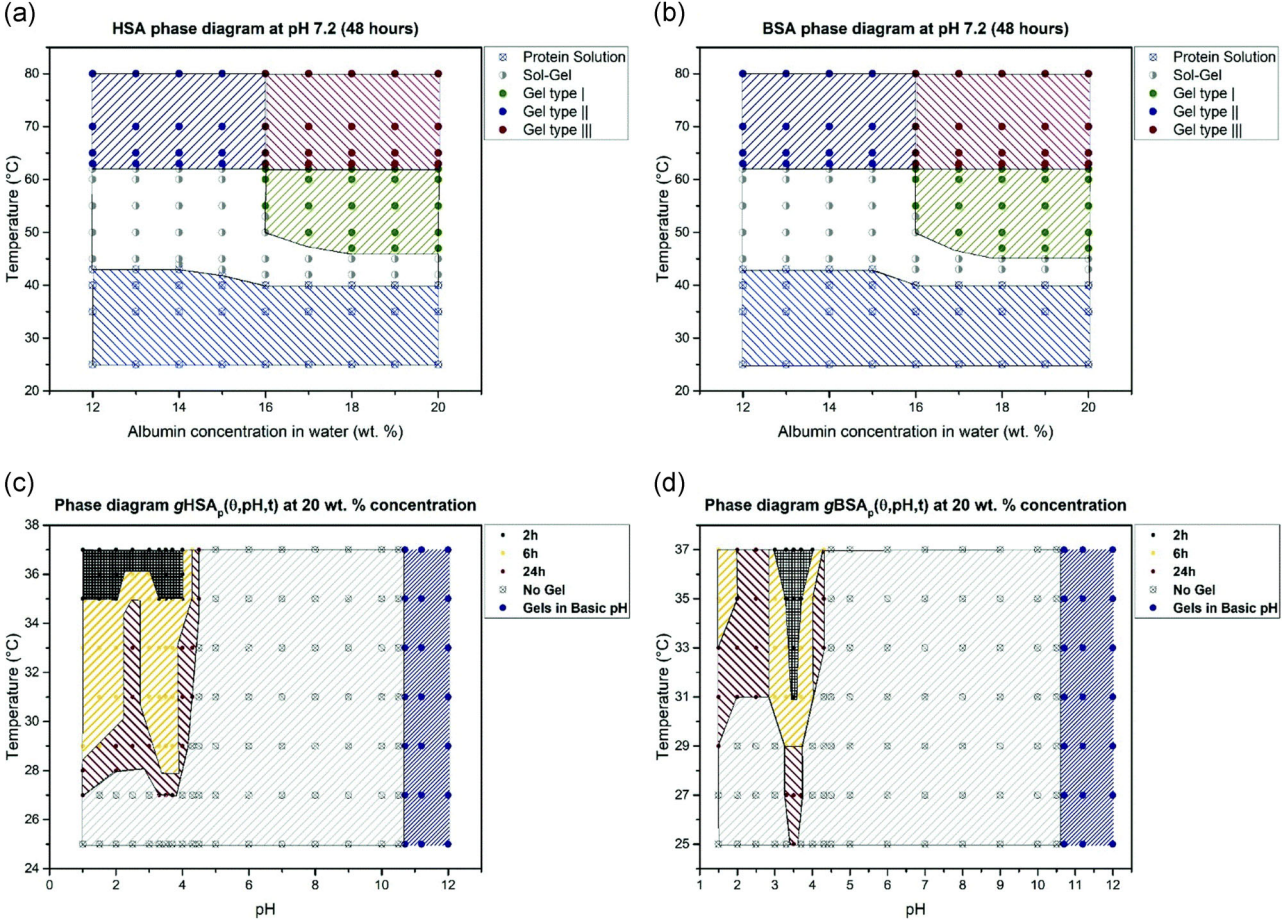
Phase diagram of (a) human serum albumin (HSA) and (b) bovine serum albumin (BSA) after 48 hr of heating, at different concentrations and at neutral pH. (c) Phase diagram for 20% w/v HSA solution at varying pH values and heating times. Gels at high pH values (pH > 10.6) form in less than 2 hr at room temperature. (d) Phase diagram for 20% w/v BSA solution at varying pH values and heating times. Image reproduced with permission from Arabi et al. (2018)

Recently, both pH and temperature‐dependent gelation behavior in BSA and HSA have been extensively studied by Arabi et al. (2018). This led to the physical characterization of both BSA and HSA hydrogels through several phase diagrams (Figure 2). Interestingly, the authors established that gelation of BSA and HSA can occur over a wide pH range and temperatures (pH 1.0–4.3 and pH > 10.6 at 37°C or pH 7.0–7.2 at 50–65°C). However, the gelling mechanism of BSA and HSA or the biocompatibility of alkali‐induced albumin hydrogels were not investigated. It is highly likely molecular and structural differences in albumin isomers exist across the different gelling conditions, and this will, in turn, affect the properties of the albumin hydrogel such as available binding sites. Further research in this area can help in the conjugation or functionalization of albumin hydrogels with target proteins in the future.

In contrast to acidic pH, albumin transitions from the N‐form isomer to the B‐form (basic form) around pH 8 then to the A‐form (aged form) around pH 10 and above (Amiri, Jankeje, & Albani, 2010; Chen et al., 2019; Leggio, Galantini, & Pavel, 2008). The A‐form isomers then form aggregates and the exact mechanism of gelation remains poorly understood (Chen et al., 2019). Recently, Chen et al. (2019) reported that alkali‐induced BSA hydrogels formed at pH 12 and 37°C incubation were mechanically stable, and exhibited self‐healing and autofluorescence properties. However, similar to acid‐induced albumin hydrogels, alkali‐induced hydrogels required neutralization with DMEM to pH 7.4. Biocompatibility of the neutralized hydrogel was subsequently demonstrated by cell culture of human lung carcinoma cells (A549 cell line) over a 48‐hr period. Unfortunately, no quantitative data on cell experiments were provided and the long term (weeks to months) in vivo stability of these alkali‐induced hydrogels remains unknown.

### 2.2 Thermally induced albumin hydrogels

Heat‐induced gelation is a more commonly reported method to obtain stable albumin hydrogels (Amdursky et al., 2018; Arabi et al., 2018; Baler et al., 2014; Nandlall et al., 2010; Peng et al., 2016). However, one important consideration is that applying high heat will cause the denaturation of albumin as its structure starts to unfold at temperatures above 65°C (Borzova et al., 2016). The higher the temperature above 65°C, the greater the degree of unfolding and aggregation. This denaturation temperature of albumin can also be lowered by changes in pH and the addition of ions or redox reagents, for example, magnesium (Haque & Aryana, 2002) and urea (González‐Jiménez & Cortijo, 2002), respectively. Another important consideration is that with the denaturation and aggregation of albumin, its binding sites for ions, drugs, and proteins can change, together with other physical properties of the albumin hydrogels such as turbidity.

pH‐neutral, thermally induced albumin hydrogels increase in turbidity as temperature, albumin concentration, and ionic content increase due to extensive denaturation of albumin molecules (Amdursky et al., 2018; Murata, Tani, Higasa, Kitabatake, & Doi, 1993). This is in stark contrast to pH‐induced albumin hydrogels which still have a clear to translucent appearance at higher albumin concentrations when incubated at room temperature to 37°C, even though denaturation still occurs. Baler et al. (2014) have demonstrated that thermally induced BSA hydrogels have larger pore sizes, a higher Young's modulus and lower degradability compared to pH‐induced BSA hydrogels, however, these properties vary with albumin concentration and more extensive characterization over a wider range of gelation conditions is needed, particularly in HSA. The tunable characteristics of thermally induced albumin hydrogels indeed make it seem appealing, but opaque or turbid hydrogels have limited usefulness in biological studies as it precludes normal brightfield microscopy. However, this may be overcome by the addition of sodium chloride. To reduce the turbidity of thermally induced albumin hydrogel, Murata et al. (1993) reported that the addition of sodium chloride into BSA solution resulted in transparent gels within a specific concentration range. Studies to determine if this effect is reproducible in HSA are still pending.

Interestingly, apart from applying high heat, it has been recently demonstrated that albumin hydrogels could be formed by salt‐induced cold gelation (Ribeiro et al., 2016). With the addition of calcium chloride and DL‐dithiothreitol to a BSA/HSA mix and heating at 60°C for 30 min, followed by cooling and freezing at −20°C for 2 days, an albumin hydrogel can be obtained. The resulting hydrogel was freeze‐dried to create a porous scaffold which was later shown to be biocompatible.

In summary, current methods to derive pH‐induced or thermally induced hydrogels have shown that the (a) albumin concentration, (b) the presence of ions or redox reagents, (c) the range of pH, (d) heating temperature, and (e) duration of heating, are all crucial factors for gelation. These, in turn, affect the final properties of the albumin hydrogel.

### 2.3 Chemically crosslinked albumin hydrogels

Chemical crosslinking is the most reported method to derive albumin hydrogels (Abbate, Kong, & Bansal, 2012; Bai et al., 2019; Feldman & McCauley, 2018; Gallego, Junquera, Meana, Álvarez‐Viejo, & Fresno, 2010; Gallego, Junquera, Meana, García, & García, 2010; He, Jean‐Francois, & Fortier, 2012; Hirose, Tachibana, & Tanabe, 2010; Kim et al., 2015; Li et al., 2014; Lisman, Butruk, Wasiak, & Ciach, 2014; Ma et al., 2016; Manokruang & Lee, 2013; Noteborn, Gao, Jesse, Kros, & Kieltyka, 2017; Oss‐Ronen & Seliktar, 2011; Overby & Feldman, 2018; Raja, Thiruselvi, Mandal, & Gnanamani, 2015; Scholz et al., 2010; Upadhyay & Rao, 2019; Zhao et al., 2019; Zhou et al., 2018). Synthetic polymers such as polyethylene glycol (PEG) are activated to form PEG‐albumin complexes (e.g., with 4‐nitrophenyl‐chloroformate), or alternatively functional groups may be added to the ends of the PEG molecule to target specific chemical compositions or binding sites of other target proteins for conjugation. For example, the methoxy‐polyethylene glycol group at the ends of each PEG‐succinimidyl propionate (PEG‐SPA), PEG‐succinimidyl succinate (SS), and PEG‐succinimidyl glutarate (PEG‐SG) molecule, are able to exchange a hydroxyl group with an *N*‐hydroxysuccinimide (NHS) group. These functionalized PEG‐NHS molecules can then form amide linkages with amino acids such as lysine from target proteins.

The configuration of the PEG backbone and the number of hydrolytically cleaved functional groups determine the overall stability of PEG‐NHS molecules. As such, PEG‐SS and PEG‐SG molecules are more easily degraded compared to PEG‐SPA as these molecules contain esters in their backbone which are affected by hydrolysis. In vivo once hydrolyzed, PEG chains are cleared mainly through the kidneys, and to a lesser extent, the liver and gut (Baumann et al., 2019). This can be taken advantage of to suit the rate of biodegradability desired. Apart from functionalization with NHS groups, PEG can also be functionalized with maleimide (PEG‐MAL) or diacrylate (PEG‐DA). The ‐MAL end group crosslinks thiol groups that are present in amino acids such as cysteine and thiolated target proteins such as thiolated albumin. PEG‐DA is activated by exposure to ultraviolet light and photo‐crosslinking ensues. However, intracellular damage from reactive oxygen species and cytotoxicity may result from prolonged or high‐intensity UV exposure (de Jager, Cockrell, & Du Plessis, 2017) if cells were encapsulated in bulk during gelation. This can be circumvented by creating a porous scaffold through sacrificial molding then seeding cells within it (Shirahama et al., 2016). Other less‐commonly used agents to crosslink albumin include glutaraldehyde (Gallego, Junquera, Meana, Álvarez‐Viejo, et al., 2010; Gallego, Junquera, Meana, García, et al., 2010; Ma et al., 2016; Upadhyay & Rao, 2019; Zhao et al., 2019), glutathione (Bai et al., 2019; Raja et al., 2015), dithiothreitol (Hirose et al., 2010), transglutaminase (Li et al., 2014), polyaminourethane (Manokruang & Lee, 2013), oxidized dextran (Lisman et al., 2014), and *N*,*N*‐methylenebisacrylamide (Abbate et al., 2012).

With PEG being the most common polymer used in the crosslinking of albumin, perhaps the greatest concern in the clinical application of PEG‐albumin hydrogels is immunogenicity. Although it is well established that albumin itself is poorly immunogenic, there is growing evidence that PEG is not bioinert. Clinical trials involving PEGylated drugs have demonstrated that the occurrence of PEG‐specific immunoglobulin M (IgM) and IgG antibodies in patients is not infrequent and it can result in reduced drug efficacy, mild to moderate immune reactions, and adverse outcomes (Zhang, Sun, Liu, & Jiang, 2016). This considered other methods of albumin gelation and conjugation should be explored.

## 3 MECHANICAL PROPERTIES

Uniaxial compression and tension, and indentation have been employed to measure Young's modulus of albumin hydrogels (Amdursky et al., 2018; Baler et al., 2014; Fleischer et al., 2014). The main concern when testing compliant materials such as hydrogels is separating inelastic (time‐dependent) and elastic characteristics as Young's modulus should be independent of time. In this context, the load and displacement measuring systems and the inertia of the testing setup are important. Often hydrogels require a customized setup suitable for low load testing rather than conventional mechanical testing set‐ups. Nanoindenters and microindenters, on the contrary, have accurate load and displacement measuring systems, but inevitably indentation is likely to generate regions of high local stress, which make inelastic deformation even more likely. Furthermore, unless a relatively large indenter tip is used, an indentation may not be suitable for property measurement as it cannot sample volumes large enough to be representative.

Baler et al. (2014) measured Young's modulus of pH‐ and thermally induced BSA hydrogels using a custom‐built flat‐ended cylindrical indenter with a radius of 0.44 mm. For a 17 wt% pH‐induced hydrogel, the values were found in the range of 3–35 kPa for pH between 3 and 4, with the highest value obtained at pH 3.5. No solid gels were formed for pH below 3 and above 4. The 20 wt% pH‐ and thermally induced hydrogels gave values of about 46 and 67 kPa, respectively. Fleischer et al. (2014) measured Young's modulus of electrospun 10% (w/v) BSA scaffolds under uniaxial tension. Scaffolds were submerged in PBS for 15 min before testing. The Young's modulus values were 1.22 ± 0.07 and 0.43 ± 0.07 MPa, respectively, for uniaxially aligned and randomly oriented albumin scaffolds, with ribbon‐like fibers. No information was provided in the loading direction. It is assumed that they measured the through‐thickness Young's modulus of the scaffolds and that the albumin fibers in both scaffolds were lying in‐plane. The randomly oriented fibrous scaffolds were reported to have a larger pore size and slightly wider albumin fibers. No information was provided on the scaffold porosity and fiber density so it is difficult to make any comparisons. Amdursky et al. (2018) measured Young's modulus of 3–9 wt% BSA hydrogels under tension. The hydrogels were not submerged in solution during testing. The Young's modulus was found to increase from ~5 to 17 kPa with increasing albumin concentration. Under confined compression, the values varied from ~0.2 to 4.4 MPa for 3–9 wt% BSA hydrogels. In all of the above studies, it would have been useful if the authors provided an expanded view of the low‐strain region used to measure Young's modulus.

Rheology tests have also been used to characterize the viscoelastic behavior of BSA hydrogels. Baler et al. (2014) investigated the gelation kinetics of both pH‐ and thermally induced BSA hydrogels at 37 and 80°C, respectively, with a 0.5% oscillatory strain. pH‐induced BSA hydrogels (pH 3.5) formed slowly (~330–2,300 s) compared to thermally induced BSA hydrogels (~20–65 s). They exhibited a lower storage modulus (*G*′) compared to thermally induced hydrogels with the same BSA concentration. The *G*′ and loss modulus (*G*″) values for both 16 and 20 wt% thermally induced BSA hydrogels reached a plateau at around 120 and 60 kPa, respectively, after 30–50 s. Amdursky et al. (2018) obtained *G*′ ~13 and *G*″ ~4 kPa for a 9 wt% thermally induced BSA hydrogel (10 Hz, 80°C and 0.5% oscillatory strain).

There is limited data on the strength and failure strains of albumin hydrogels (Amdursky et al., 2018; Zhou et al., 2018). Zhou et al. (2018) reported tensile strengths of about 40 kPa for both a 20 wt% HSA hydrogel and a 0.5% bioglass‐activated/HSA composite hydrogel. Amdursky et al. (2018) measured tensile failure strains between 35% and 100% for 3–9 wt% BSA hydrogels. The stress‐strain curves suggest that increasing BSA concentration does not have a strong effect on the measured fracture strengths, which are a few kPa, whereas failure strains tend to decrease markedly reducing the toughness of the hydrogels.

## 4 BIOLOGICAL EFFECTS AND USES

Albumin is confined mainly to the vascular and interstitial space within the human body. It binds to nine different cell surface receptors and is relatively inert to many cell types (Merlot, Kalinowski, & Richardson, 2014). Several binding sites on albumin allow the attachment of important molecules, proteins, and ions which in turn provides stability in solution. It is, therefore, used commonly in cell culture media as a carrier protein, however, albumin alone in its normal form (N‐form) has rarely been used as a culture matrix because of limited cell attachment in 2D cultures (Hirose et al., 2010). Several groups have overcome this problem successfully by functionalizing albumin hydrogels with fibronectin (Amdursky et al., 2018), laminin (Fleischer et al., 2014), or culturing cells in 3D hydrogels with crosslinked or denatured albumin. Below, studies grouped by experimental cell or tissue types are discussed.

### 4.1 Bone and cartilage regeneration

Two separate studies (Gallego, Junquera, Meana, Álvarez‐Viejo, et al., 2010; Gallego, Junquera, Meana, García, et al., 2010) isolated human osteoblasts from teeth (third mandibular molars) and cultured these cells in HSA enriched media. An albumin‐rich gel was then created from patient‐derived serum by first crosslinking with glutaraldehyde. Further freezing at −70°C overnight and dehydration with ethanol created a porous scaffold which was later seeded with osteoblasts. These constructs were transplanted beneath the skin of immunodeficient mice. Osteoblast proliferation was reported both in vitro and in vivo. After 75–150 days, analysis of transplanted constructs demonstrated the deposition of human vimentin, osteocalcin, calcium, and phosphate matrix along with bone within the pores of the scaffold. Here the significance of vimentin positivity is unclear. Vimentin inhibits osteoblastic differentiation, but the deposition of bone matrix by mature osteoblasts was reported. It is also important to note that the albumin‐rich scaffolds were derived from human serum, which may contain other proteins and growth factors that were not removed by the gelation and drying process. Therefore, it is not possible to attribute any biological effects observed in this study solely to albumin although there is evidence in the literature that albumin itself encourages osteoblast proliferation (Ishida & Yamaguchi, 2004).

Apart from bone‐forming cells (osteoblasts), chondrocytes derived from human articular cartilage were able to proliferate in a PEGylated albumin hydrogel supplemented with hyaluronic acid (Scholz et al., 2010). It was reported that cells cultured within this hydrogel had a characteristic gene signature for aggrecan, collagen type I and type II. Unfortunately, with the presence of three polymers in the hydrogel, it is not discernible what the actual effects of albumin are. Nonetheless, it serves its function as a biocompatible scaffold.

Interestingly, Li et al. (2014) reported that freeze‐drying an albumin gel crosslinked with transglutaminase produced a scaffold with physical and mechanical properties similar to collagen scaffolds. More significantly, the authors were able to successfully differentiate human mesenchymal stem cells (MSCs) seeded in these scaffolds into osteoblasts, demonstrating the potential of such a scaffold for bone tissue engineering and regenerative medicine. However, one important limitation is that these scaffolds were made with BSA and further research is needed to determine if this is reproducible with HSA hydrogels for future clinical applications.

### 4.2 Skin regeneration and wound healing

Feldman and McCauley (2018) reported that a species‐specific, albumin hydrogel scaffold could accelerate the epithelialization rate of full‐thickness wounds after 2 weeks. This effect was further augmented with the introduction of MSCs expressing transforming growth factor β3 (TGFβ_3_) in the bulk of the scaffold. These experiments were conducted using albumin derived from rabbits and transplanted into immunocompetent rabbits, however, no significant differences were noticed when comparing the overall rate of wound healing with control groups. Admittedly, the study was also underpowered to detect an effect. Zhou et al. (2018) created a composite albumin hydrogel by crosslinking HSA with PEG‐SS_2_. Bioglass was added to increase the gelation time of the composite gel and to allow the delivery of calcium and silicon ions at the site of injury after injection of the acellular hydrogel. The authors demonstrated wound healing, measured by epidermal thickness, dermal thickness, and angiogenesis, were significantly increased by acellular HSA‐PEG‐SS_2_ hydrogels, but the greatest effect was observed in the composite hydrogel with Bioglass (HSA‐PEG‐SS_2_‐Bioglass) at 14 days. It is noteworthy that an immunodeficient mouse model was used (BALB/c nude). As such, the effect of the HSA‐PEG‐SH_2_‐Bioglass hydrogel in the presence of a competent immune system is not known and this may conceal immune‐mediated reactions or detrimental effects on wound healing in normal test subjects.

Apart from human MSCs, human skin fibroblasts (BJ‐5ta; Ribeiro et al., 2016) and mouse adipose fibroblasts (L929; Hirose et al., 2010; Lisman et al., 2014) have also been cultured successfully on albumin hydrogels but these were used only in the context of cytotoxicity testing.

### 4.3 Lung & breast

Cell survival studies have been performed successfully with lung cancer cell lines A549 (Bodenberger, Kubiczek, & Rosenau, 2017; Chen et al., 2016; Ma et al., 2016), and breast cancer cell lines MCF‐7 (Bodenberger et al., 2017) and ZR75‐1 (Nandlall et al., 2010). However, the use of albumin hydrogels in lung and breast tissue engineering as well as regenerative medicine is limited.

### 4.4 Heart

**Figure 3:**
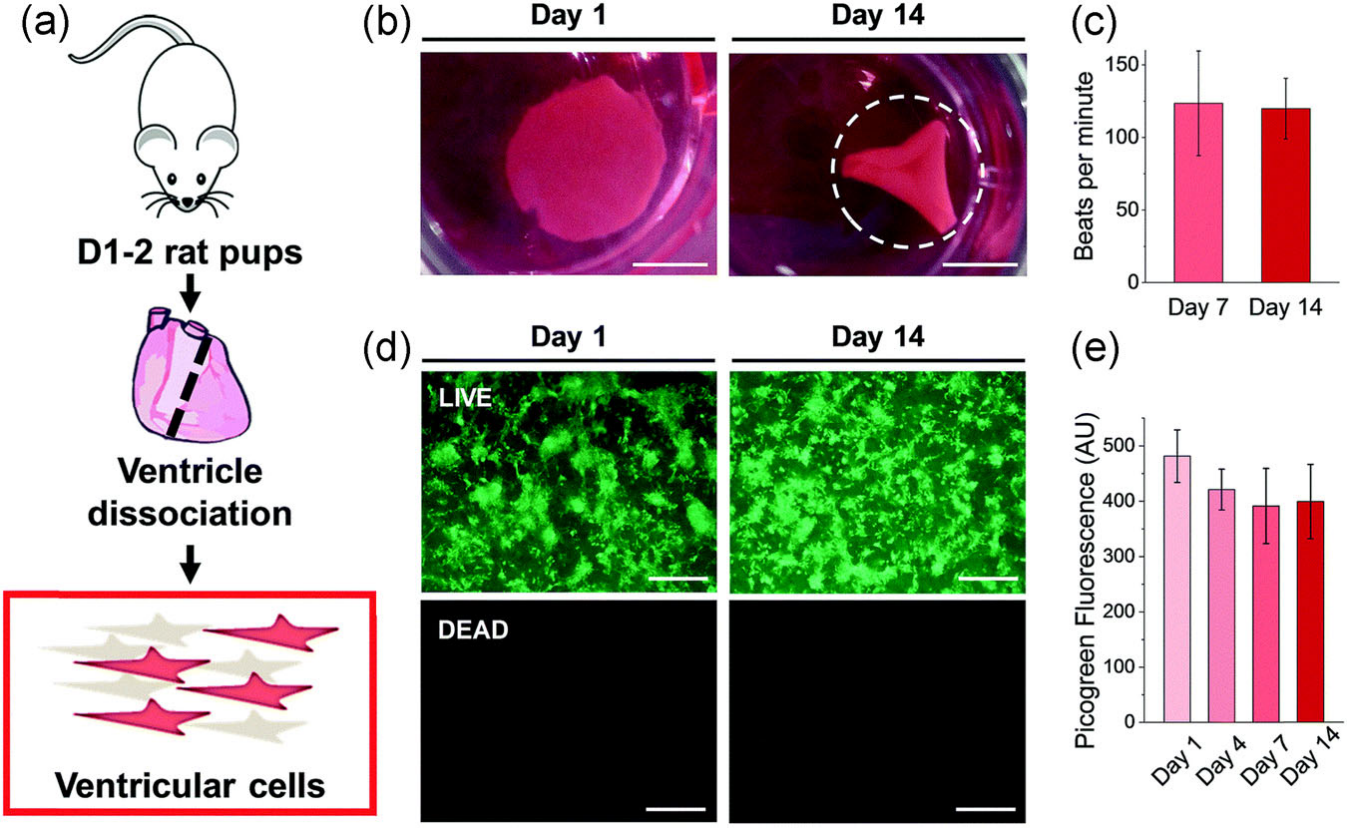
(a) Isolation of neonatal rat ventricular cardiomyocytes. (b) Bovine serum albumin hydrogel construct had folded spontaneously at Day 14 to create a three‐dimensional environment. (c) No differences in cardiomyocytes function (beats/min) were noticed at Days 7 and 14 (*p* > .05). Good cell survival was demonstrated over 2 weeks by (d) Live/Dead staining and (e) Picogreen double‐stranded DNA quantification. Image reproduced with permission from Amdursky et al. (2018)

Albumin hydrogels have been reported to enhance the functionality of neonatal rat ventricular cardiomyocytes (NVRM) and cardiomyocytes derived from human‐induced pluripotent stem cells (hiPSC‐CMs; Humphrey et al., 2017). Humphrey et al. (2017) reported albumin hydrogels had a positive effect on calcium handling (time to peak and rate of decay) in NVRM and hiPSC‐CMs. The authors used glass as a negative control; however, positive control with an alternative matrix was missing. It is, therefore, not possible to discern what the effects of albumin hydrogels are in comparison to physiological standards. Amdursky et al. (2018) reported that NVRM cultured on a pH‐induced albumin hydrogel and functionalized with fibronectin, produced NVRM with gene profiles (Myh7, Myh6, Myl2, Actn2, Tnnt2, Acta2, SERCA2, Atp2a2, Slc8a1, Pln, and Ryr2) closely resembling that of freshly isolated cardiomyocytes, whereas NVRMs cultured on glass alone began to de‐differentiate. Furthermore, co‐culture of NVRM with rat endothelial cells, smooth muscle cells, and fibroblasts on the surface of the hydrogel resulted in contractile cardiac tissue which could be paced by external electrical stimulation (Figure 3). Interestingly, neutralization of the acidic albumin hydrogel by leaching with neutral pH media was not performed and the effect of the albumin hydrogel acidity (pH 2) on NVRM is not known.

Fleischer et al. (2014) created an electrospun scaffold from albumin hydrogels crosslinked by trifluoroethanol (TFE) and β‐mercaptoethanol (BME). Interestingly, the authors reported that NVRM proliferated, self‐organized, and formed cardiac tissue in these albumin scaffolds when functionalized with laminin. Furthermore, indices of cardiac function; the rate of contractility and amplitude, were significantly enhanced compared to scaffolds made from polycaprolactone (PCL). However, it is important to note that the laminin was applied by coating the albumin scaffolds with fetal bovine serum (FBS) instead of pure laminin alone. This suggests other soluble proteins and growth factors in FBS could also be present on the fibers and not just serum laminin alone. Also, the control group with PCL scaffolds were coated with fibronectin instead of FBS so a fair comparison cannot be made.

### 4.5 Liver

There is limited research on albumin hydrogels in liver tissue engineering and regenerative medicine. Zhao et al. (2019) created a ruthenium‐albumin hydrogel crosslinked by glutaraldehyde and reported cell survival of both liver cancer cell line HepG2 and normal human fetal hepatocyte cell line L02. The survival rates of HepG2 cells decreased with increasing concentrations of ruthenium but this was an intended effect.

### 4.6 Nerves

Albumin scaffolds promoting the proliferation, differentiation, and branching of human iPSC‐derived neural stem cells (hiPSC‐NSC) was reported by Hsu, Serio, Amdursky, Besnard, & Stevens (2018). An electrospun scaffold was created from albumin hydrogels crosslinked by TFE and BME, then coated with hemin, laminin and basic fibroblast growth factor. hiPSC‐NSCs seeded on uncoated albumin scaffolds were observed to have significantly high death rates. Oddly, the cell death rates on both coated and uncoated albumin scaffolds were similar. In contrast, cell death rates on uncoated glass (negative control) were significantly lower. More Ki67‐positive cells were also observed on uncoated glass than on coated scaffolds although there were more β3‐tubulin‐positive cells in coated scaffolds. Neurite branching was only observed to be more significant than the negative controls when an electrical stimulus was applied. Given the mixed results, further investigation is needed in this area.

### 4.7 Drug delivery

The role of albumin molecules in drug delivery is well established, however research in albumin hydrogels for controlled drug release and delivery is still growing. Kim et al. (2015) utilized a PEG‐HSA hydrogel loaded with an apoptotic TRAIL protein to successfully induce cancer cell death and reduce tumor size in a murine model injected with a pancreatic cancer cell line (Mia Paca‐2). Successful controlled drug release was also demonstrated using a composite hydrogel (Dextran‐HSA‐PEG) loaded with anticancer drug doxorubicin to eliminate breast cancer cells (MCF‐7) in vitro (Noteborn et al., 2017). More recently, Zhao et al. (2019) demonstrated the ability of albumin hydrogels to selectively deliver metal ions to liver cancer cells (HepG2) for anticancer therapy or imaging.

## 5 BIODEGRADABILITY

The biodegradability of albumin hydrogels depends on the way the albumin hydrogel is synthesized. Baler et al. (2014) demonstrated that albumin hydrogels formed by electrostatic self‐assembly in acidic pH were easily degradable in vitro and in vivo, whereas thermally induced albumin hydrogels were resistant to degradation. In vitro, an 8 M solution of urea degraded acid‐induced albumin hydrogels within 17 hr, whereas in vivo degradation occurred in an immunocompetent rat model (Sprague–Dawley) over 4 weeks with little evidence of inflammation and the site of transplantation. In contrast, thermally induced albumin hydrogels were resistant to chemical and physiological degradation. Thermally induced BSA hydrogels were still intact 4 weeks post‐transplantation and a fibrous capsule around the scaffold was noted. Interestingly, local inflammation was noted when untreated BSA was injected but this resolved with time.

Albumin hydrogels derived by glutaraldehyde‐induced crosslinking seem to exhibit poor biodegradability and local immunogenicity. (Gallego, Junquera, Meana, Álvarez‐Viejo et al., 2010) reported that glutaraldehyde‐crosslinked HSA hydrogels, when transplanted in an immunodeficient mouse model, remained partially degraded at 150 days. Calcification of the scaffolds and injury to overlying skin were also noted. Ma et al. (2016) reported hyperkeratosis in all mice after the injection of glutaraldehyde crosslinked BSA hydrogels but complete degradation after 2 months. In one out of two test subjects, inflammation was noted in the surrounding skin and a fibrous capsule around the BSA hydrogel was developing. The strain of mice used was immunodeficient. The crosslinking process could account for the skin reactions as other methods of gelation, for example, electrostatic self‐assembly, did not produce the same effect. The difference in degradation times could also be explained by the inherent differences between HSA and BSA although further studies are needed to confirm this.

BSA hydrogels synthesized by glutathione‐mediated oxidative refolding (Raja et al., 2015) produce less of an immunogenic response compared to BSA hydrogels synthesized by glutaraldehyde‐induced crosslinking. When transplanted into an immunocompetent rat model (Wistar), these hydrogels did not precipitate skin reactions. No obvious signs of inflammation were noted but the formation of a fibrous capsule around the hydrogel persisted in a dose‐dependent manner, that is, higher albumin concentrations were associated with thicker fibrous capsules. Degradation times also increased with increasing albumin concentrations, for example, 15–20 days for 300 µM gels and 30–40 days for 600 µM gels. It would be both important and useful for future studies to determine if immunocompetency in animal models accelerates the rate of hydrogel degradation.

**Figure 4:**
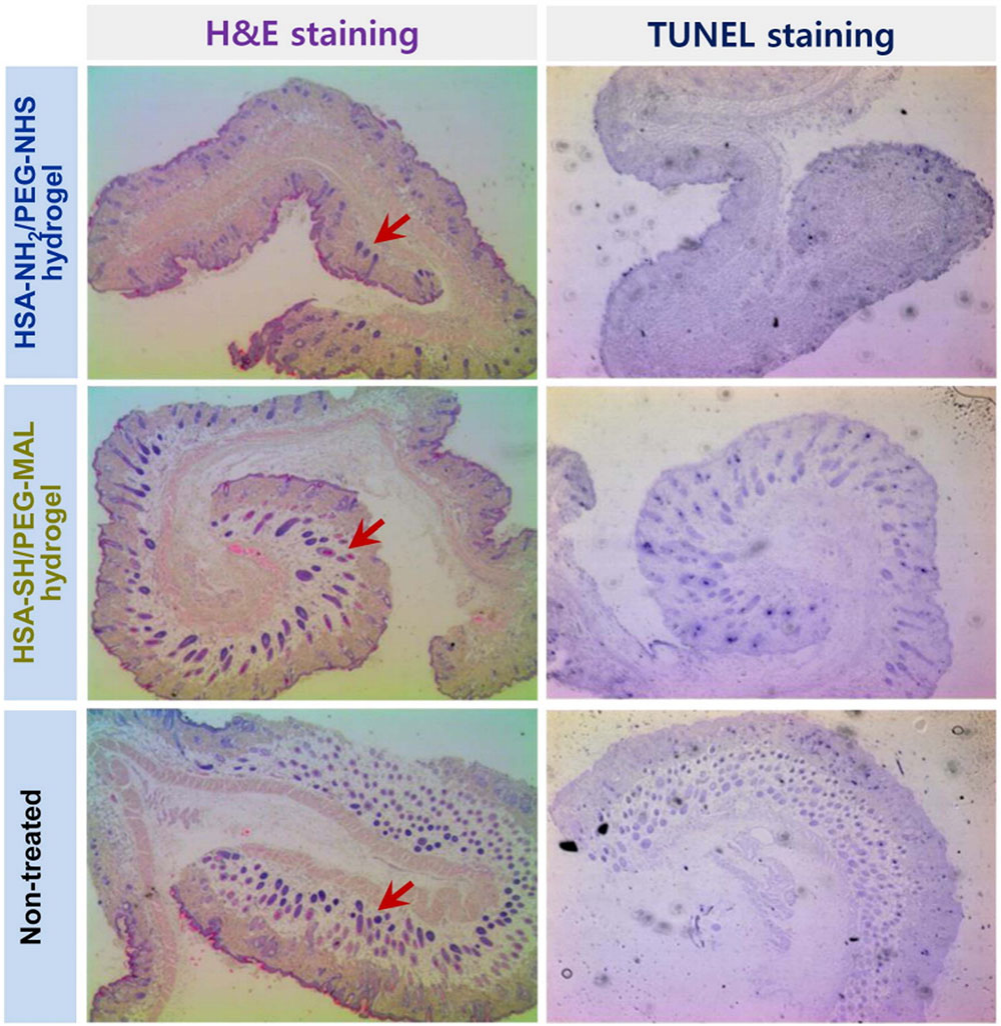
Cross‐sections of epidermal tissue at the site of transplantation of HSA‐PEG hydrogels demonstrated no evidence of inflammation or apoptosis TUNEL negative. H&E, hematoxylin and eosin; HSA‐PEG, human serum albumunr–poly(ethylene glycol); MAL, maleimide; NHS, N‐hydroxysuccinimide; TUNEL, terminal deoxynucleotidyl transferase dUTP Nick‐End Labeling, an assay for apoptosis). Image reproduced with permission from Kim et al. (2015)

PEG‐albumin hydrogels generally have a more predictable degradation period of approximately 2 to 4 weeks with no local side effects. An HSA‐SH/PEG‐MAL hydrogel was reduced to 28% of its initial weight after 21 days in an immunodeficient mouse model (Figure 4; Kim et al., 2015). Interestingly, only one study to date has created albumin hydrogels derived from species‐specific serum for in vivo experimentation. Feldman and McCauley (2018) created an albumin‐(PEGSG_2_)‐TGFβ_3,_ hydrogel scaffold from rabbit albumin and transplanted these into immunocompetent rabbits. The degradation time was reported to be 2 weeks with no immunogenic complications observed. In contrast to the above, hydrogels created from the conjugation of BSA with PEG‐derived poly‐amino‐urethane showed poor degradability after 3 weeks in immunocompetent rats (Manokruang & Lee, 2013). Unfortunately, local effects in the surrounding skin were not assessed.

## 6 COST

Animal‐derived albumin is inexpensive; however, all hydrogels should ideally be xeno‐free for clinical utility. Human albumin is considerably more expensive than animal‐derived albumin but relatively cheaper compared to other substrates used in regenerative medicine. For example, Matrigel costs approximately £1,933.33/g (Sigma‐Aldrich) and rat tail collagen‐1 costs £4,810.00/g (Sigma‐Aldrich); whereas human albumin costs £20.30/g (Sigma‐Aldrich). Other preparations of albumin such as 20% human albumin solution may be procured at cheaper and larger volumes, for example, 20 g for £54 (Octapharma Ltd).

Apart from albumin, additional costs may also be incurred by the reagents used to induce gelation of albumin and chemically crosslink target proteins. For example, hydrogel synthesized by glutaraldehyde (£ 3.60/ml for 70% glutaraldehyde, Sigma‐Aldrich) is considerably cheaper compared to functionalized PEG (£ 108.12/g for 4‐arm 10 K PEG‐SG; Creative PEGWorks). However as discussed above, albumin hydrogels crosslinked by glutaraldehyde or glutathione have a propensity to be immunogenic. As cell attachment to N‐form albumin is generally poor and conjugation of target proteins with PEG costly and laborious, better methods of functionalizing HSA hydrogels are needed.

## 7 CONCLUSION

There is a broad scope for further exploitation of albumin hydrogels in regenerative medicine. In the study of the lung, the creation of macroporous albumin scaffolds from hydrogels could be useful in engineering of lung parenchyma. Pore size and thickness could be tuned to recapitulate the alveolar space onto which lung stem cells and auxiliary cell types could be seeded. This offers an alternative to decellularized animal scaffolds and lung organoid biology could be studied in xeno‐free conditions. In regenerative cardiology, the growth of contractile heart organoids on HSA has not yet been demonstrated but remains an attractive area of research to pursue. Xeno‐free, injectable HSA hydrogels could then be a viable method of delivering cardiac stem cells or cardiomyocytes directly into injured myocardium. In regenerative hepatology, a similar approach to a cell or tissue delivery could be adopted to transplant hepatic stem cells, hepatocytes, or organoids in liver failure. However, albumin, an important marker of synthetic liver function, is released by the degradation of HSA hydrogels which may make albumin ELISAs (enzyme‐linked immunosorbent assays) difficult to interpret, particularly in animal models. In regenerative neurology, studies to determine if HSA hydrogels enhance proliferation, differentiation, and branching of hiPSC‐NSC are needed as these were previously reported in BSA.

In summary, apart from drug delivery, albumin hydrogels hold great potential as a biomaterial for 3D cell culture, platform for cell delivery and scaffold for tissue transplantation. The inertness, poor immunogenicity, biodegradability, cost, and possibility to derive patient‐specific albumin make albumin hydrogels useful in regenerative medicine and tissue engineering. However, these have not been fully exploited and better methods of synthesizing and functionalizing albumin hydrogels are needed.
